# Prospective associations between psychosomatic complaints in adolescence and depression and anxiety symptoms in young adulthood: A Swedish national cohort study

**DOI:** 10.1016/j.ssmph.2023.101509

**Published:** 2023-09-04

**Authors:** Karina Grigorian, Viveca Östberg, Jonas Raninen, Johan Åhlén, Sara Brolin Låftman

**Affiliations:** a Department of Public Health Sciences, Stockholm University, Sweden; b Department of Clinical Neuroscience, Karolinska Institutet, Sweden; c Centre for Alcohol Policy Research, La Trobe University, Melbourne, Australia; d Centre for Epidemiology and Community Medicine, Stockholm County Council, Stockholm, Sweden; e Department of Global Public Health, Karolinska Institutet, Sweden

**Keywords:** Psychosomatic complaints, Adolescents, Depression, Anxiety, Self-report, Longitudinal

## Abstract

**Background:**

Psychosomatic complaints are reported by high shares of adolescents in Sweden and elsewhere. Yet, little is known about to the extent to which the frequency, number, and persistence of such complaints in adolescence are associated with subsequent mental health problems. The aim of this study was to examine how the frequency, number, and persistence of psychosomatic complaints in middle and late adolescence are associated with depression and anxiety symptoms in young adulthood.

**Methods:**

A Swedish national cohort study of adolescents who were surveyed in 2017 (t1; age 15–16), in 2019 (t2; age 17–18) and in 2022 (t3; age 20–21 years) was used. Psychosomatic complaints were measured by questions on stomach ache, headache and difficulties falling asleep at t1 and t2. Depression and anxiety symptoms were measured by the Patient Health Questionnaire-4 (PHQ-4) at t3. Multivariable binary logistic regression analyses stratified by gender were based on data from t1, t2 and t3 (n = 2779).

**Results:**

The frequency, number, and persistence of psychosomatic complaints during adolescence were associated with symptoms of depression and anxiety in young adulthood. Both earlier (at t1 only) and more recent (at t2 only) complaints were linked to subsequent depression and anxiety symptoms, while persistent (at both t1 and t2) psychosomatic complaints showed stronger associations in girls.

**Conclusions:**

Psychosomatic complaints in adolescence were associated with depression and anxiety symptoms in young adulthood. This was true for the frequency, number, and persistence of psychosomatic complaints. Among girls, those who reported persistent psychosomatic complaints from middle to late adolescence had the highest likelihood of reporting subsequent depression and anxiety symptoms. Taken together, the results indicate that psychosomatic complaints during adolescence can translate into later depression and anxiety symptoms. Furthermore, repeated measurements of psychosomatic complaints can be used to identify the most vulnerable group.

## Highlights

• Psychosomatic complaints in mid- and late adolescence were linked to depression and anxiety symptoms in young adulthood.
• This was true for different operationalisations (the frequency, number, and persistence) of psychosomatic complaints.
• Adolescents with complaints at one time point only had an increased likelihood of later depression and anxiety symptoms.
• Girls with complaints at two time points showed the strongest association with later depression and anxiety symptoms.
• Adolescent psychosomatic complaints should not be disregarded as trivial.

## 1 Introduction

Psychosomatic complaints, such as headache, stomach ache and difficulties falling asleep, are reported by high shares of adolescents in Sweden (Public Health Agency of Sweden, 2023) and elsewhere (Inchley et al., 2020). These complaints have been seen as indicators of psychosocial stress and stressful life events (e.g., Corell et al., 2022; Garnow et al., 2021; Greene & Walker, 1997; Hjern et al., 2008; Villalonga-Olives et al., 2011). Previous studies have shown that psychosomatic complaints in adolescents reflect general stress and they can therefore be regarded as stress-related (Corell et al., 2022; Wiklund et al., 2012). There is a gender gap to the disadvantage of girls, which increases with age (Torsheim et al., 2006; Ravens-Sieberer et al., 2009; Friberg et al., 2012; Inchley et al., 2020; Public Health Agency of Sweden, 2023). Studies have demonstrated an increase in this type of complaints during recent decades and especially among girls, both in Sweden (Hagquist et al., 2019; Public Health Agency of Sweden, 2023) and in other countries (Hagquist et al., 2019; Potrebny et al., 2019). The prevalence and the increase are higher among adolescents in Sweden than in other Nordic countries (Hagquist et al., 2019). In an international perspective, the time trends have shown to differ across countries (Cosma et al., 2020) and the observed general increase is relatively small (Potrebny et al., 2017).

The prevalence of psychosomatic complaints is highly dependent on the chosen operationalisation, and the reported complaints may be temporary. Nevertheless, previous studies have shown that psychosomatic complaints can be indicators of future problems, including mental health outcomes. Two studies based on data from a cohort from Uppsala, Sweden, showed that somatic complaints measured at age 16–17 predicted diagnoses of depression and anxiety in adulthood (Bohman et al., 2012) as well as hospitalisation for psychiatric disorders (Bohman et al., 2018). In their study based on Swedish regional cohort data, Giannotta et al. (2022) presented prospective associations between psychosomatic complaints in adolescence at one time-point (in two cohorts of adolescents, 13 and 15 years old) and self-reported depression and anxiety three and six years later. Additionally, Shelby et al. (2013) presented longitudinal links between abdominal pain in childhood and anxiety in young adulthood in a study based on US data. Very few studies have examined the significance of temporary vs. persistent psychosomatic complaints in relation to later mental health outcomes. One exception is the study by Shanahan et al. (2015), which used data from the American Great Smokey Mountains Study to investigate the associations between somatic complaints from several measurement points at ages 9–16 and mental health outcomes at ages 19–26. This study showed that both frequent and persistent somatic complaints in childhood and adolescence were associated with an increased risk of depression and anxiety in young adulthood, and that individuals with persistent complaints across developmental periods had a particularly high risk (Shanahan et al., 2015).

While several of the studies listed above have examined psychosomatic complaints during childhood and/or the period from early to middle adolescence, the developmental stage of late adolescence has largely been overlooked. The period from middle to late adolescence typically denotes important transitions, such as the entry to upper secondary school, and during late adolescence individuals plan for their future education and work career and assume a more active role in their own development (Zarrett & Eccles, 2006). Alongside important developmental and social changes, this period is notable for frequent mental health problems, as well as increased experiences of negative emotions (Frost et al., 2015; Larson et al., 2002),a Wiklund, 2012and sadnesGarnow et al., 2021s. Furthermore, young adulthood is also frequently marked by mental disorders, which are accompanied by a considerably increased risk of mental health problems later in life (Gustavson et al., 2018). Therefore, these developmental periods represent a particular interest for studying psychosomatic complaints and their association with symptoms of mental disorders.In additione, there is a scarcity of research focusing on persistent psychosomatic complaints (reported at multiple time points). The data we utilise in the current study enables us to track individuals from middle and late adolescence to young adulthood. To the best of our knowledge, the prospective links between persistent psychosomatic complaints in middle and late adolescence and subsequent depression and anxiety symptoms in young adulthood have not yet been examined.

In addition to the longitudinal nature of the data, the exploration of different measures of psychosomatic complaints (including frequency, number, and persistence) enables us to contribute to the ongoing scholarly discussion about what adolescent psychosomatic complaints represent. It also helps us ascertain if it is feasible to distinguish between varying levels of severity. In a study based on interviews with forty-one Swedish 15-year-olds, Wickström & Lindholm (2020) found that while certain adolescents regarded psychosomatic complaints as deep-seated problems, others saw them rather as responses to everyday challenges. The authors concluded that if psychosomatic complaints are referred to as indicators of poor mental health, there is a risk of pathologisation of normal reactions to challenging everyday life conditions. In response to this study, Högberg et al. (2023) investigated whether a bimodal pattern was to be found also in population-based surveys. In their analyses of self-reported psychosomatic complaints from eight rounds of the cross-sectional Swedish Health Behaviour in School-aged Children study, they found no evidence of any bimodal distributions of psychosomatic complaints into ‘trivial’ and ‘more severe’ levels of complaints. Instead, the distribution of psychosomatic complaints scores based on the number and frequencies of complaints showed a continuum with no distinct clusters of respondents (Högberg et al., 2023). Another approach to address the inquiry of whether reports of psychosomatic complaints can be classified as ‘trivial’ or ‘severe’ involves utilising population-based surveys to analyse the associations that psychosomatic complaints might share with subsequent mental health conditions. Specifically, this involves to explore whether, and if so how, these associations vary based on how psychosomatic complaints are defined and measured.

The current study focuses on psychosomatic complaints during mid- and late adolescence using different operationalisations, and analyses potential associations with depression and anxiety symptoms in young adulthood. Given the clear and consistent female excess in adolescent psychosomatic complaints – which may partly be understood by sex differences in biology (Barsky et al., 2001), but also by social constructions of masculinities and femininities (Barsky et al., 2001; Landstedt & Gillander Gådin, 2021; Mayor, 2015) – it is relevant to examine potential gender differences in the prevalence and persistence of psychosomatic complaints across adolescence, and if the association between adolescent psychosomatic complaints and subsequent mental health problems differs by gender. Since girls tend to report more psychosomatic complaints (Aanesen et al., 2017; Högberg et al., 2020; Ravens-Sieberer et al., 2009; Sumter & Baumgartner, 2017; Sweeting et al., 2007; Torsheim et al., 2006) and depression and anxiety (e.g., Giannotta et al., 2022) than boys, gender stratified analyses were performed throughout. In addition, differences by gender were examined with interaction terms in analyses of the total sample.

Therefore, the aim of the current study is to examine if the frequency, number, and persistence of psychosomatic complaints in boys and girls across middle and late adolescence are associated with depression and anxiety symptoms in young adulthood.

## 2 Material and methods

### 2.1 Study design and material

The data was derived from Futura01, which is a national Swedish cohort study of adolescents attending grade 9 in 2017 (∼15–16 years). The first data collection was carried out as a classroom questionnaire during the first half of 2017. The second wave was performed in 2019 (when respondents typically attended the second grade of upper secondary school; ∼17–18 years) as a web survey or postal survey. The third wave was performed in 2022 (∼20–21 years) as a web survey.

For the first wave (t1), 500 schools (and one class in each school) were randomly selected with 343 schools accepting to participate (69%). No statistically significant differences were found between participating and non-participating schools regarding grade point average, the proportion of highly educated parents, or the proportion of parents with a foreign background (Raninen, 2020). On the day of the baseline study, a total of 6769 adolescents were present in school and 5722 agreed to participate (85%); the final sample for t1 comprised of 5537 respondents. In the second wave (t2), 4141 of those responding at t1 agreed to participate (75%). The majority of participants at t2 completed the questionnaire as a web survey (83%) and a smaller portion filled in a postal questionnaire (17%). In the third wave (t3), 2956 of those who responded at both t1 and t2 (53% of the t1 sample or 71% of the t2 sample) agreed to participate.

**Fig. 1:**
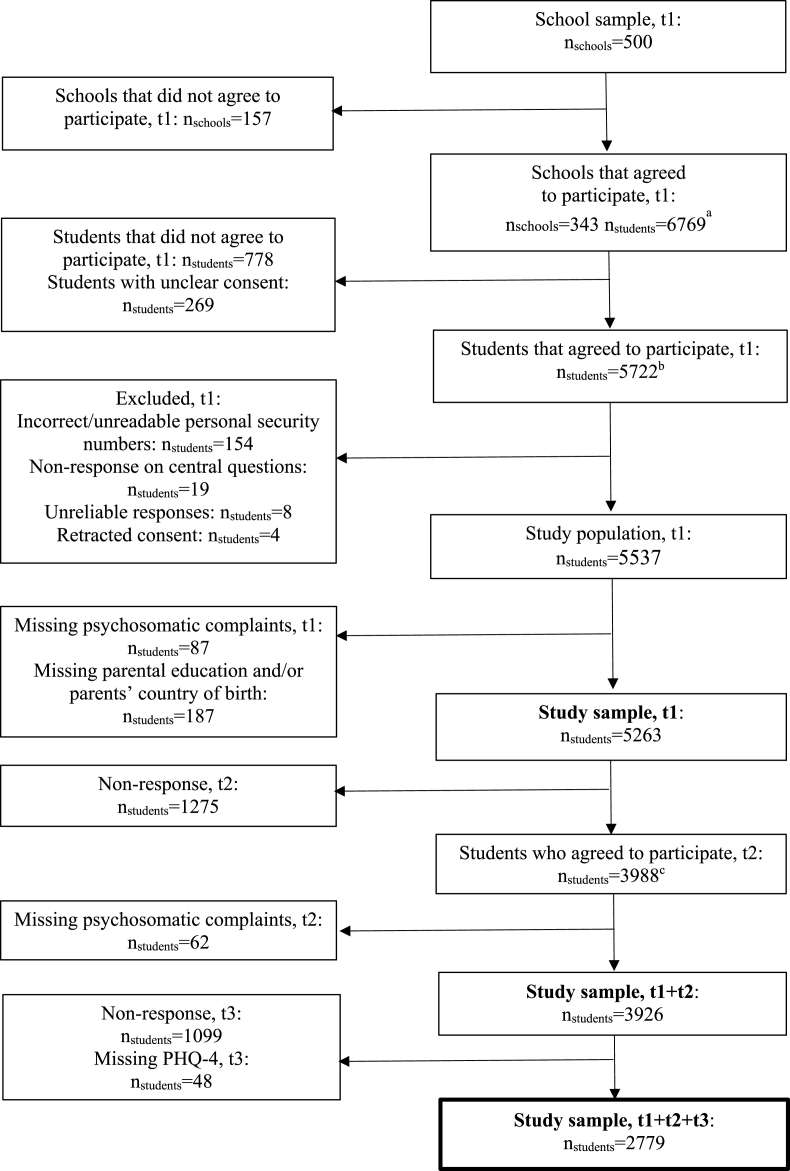
Flow chart of the study sample. ^a^ Present at school on the day of the classroom survey. ^b^ Responded to the classroom survey. ^c^ Responded to the web survey (83%) or the postal survey (17%).

In the current study, we used the three waves study sample (n = 2779) that included those who participated at all time-points and did not have missing information on the variables of interest. A detailed description of the study sample is provided in Fig. 1. Information on the respondents’ parents (i.e., educational level and country of birth) has been added to the data by a linkage to administrative registers.

### 2.2 Measures

*Psychosomatic complaints (t1 and t2)* were measured by self-report questionnaires and the question: “During the past six months, how often have you had …” and the items “Stomach ache”, “Headache”, and “Difficulties falling asleep”. For all items, the response categories were “Every day”, “A few times a week”, “Once a week”, “Some time a month”, and “Less often or never”. These items have been used previously to measure psychosomatic complaints (Låftman & Magnusson, 2017; Låftman & Östberg, 2006; Magnusson & Låftman, 2019; Östberg et al., 2006). The three psychosomatic complaints were moderately correlated: the strongest correlation was observed between stomach ache and headache (t1: r = 0.48; t2: r = 0.45), followed by the correlation between difficulties falling asleep and headache (t1: r = 0.37; t2: r = 0.34), and difficulties falling asleep and stomach ache (t1: r = 0.33; t2: r = 0.30).

*Depression and anxiety symptoms (t3)* were measured by the Patient Health Questionnaire-4 (PHQ-4) which is a brief screening scale for depression and anxiety, consisting of the Patient Health Questionnaire-2 (PHQ-2) which screens for depression and the Generalized Anxiety Disorder-2 (GAD-2) which screens for anxiety (Kroenke et al., 2009). The question reads: “Over the last 2 weeks, how often have you been bothered by any of the following problems?” The items were: a) “Feeling nervous, anxious or on edge”; b) “Not being able to stop or control worrying”; c) “Little interest or pleasure in doing things”; and d) “Feeling down, depressed, or hopeless”. The response categories were “Not at all” (0); “Several days” (1); “More than half the days” (2); and “Nearly every day” (3). PHQ-2 consists of items c) and d) (core criteria for depression) and GAD-2 of items a) and b) (core criteria for anxiety). The values for each pair of items were summed together to a scale ranging 0–6, and for both PHQ-2 and GAD-2, 3 was chosen as cutoff point (Löwe et al., 2010). Validity has been shown to be good for PHQ-2 (Kroenke et al., 2003; Löwe et al., 2005) and GAD-2 (Kroenke et al., 2007). The internal consistency of the scales was acceptable in the studied sample (PHQ-2: Cronbach's alpha: all = 0.67, males = 0.65, females = 0.69; GAD-2: Cronbach's alpha: all = 0.85, males = 0.82, females = 0.85), although PHQ-2 showed lower reliability coefficients than in previous studies conducted on population samples (Löwe et al., 2010; Wicke et al., 2022).

*Gender* was based on information from the participants’ personal security number. Although information about sex was drawn from register data, it is impossible to make a clear distinction between sex and gender with the nature of data we utilise. Furthermore, since biological factors (attributed to biologically given sex) and social factors (attributed to gender constructed by society) tend to interact, we assume that the measure reflects aspects associated with both sex and gender.

*Family type* was measured at t1 by the question “How do you live?” with the response categories “Lives with mother and father”; “Lives with mother”; “Lives with father”; “Lives about half of the time with mother and half of the time with father (shared residence)”, and “Other”. Four categories were formed: two parents in the same household, one parent, shared residence, and other.

*Parental education* was based on register information on father's and mother's educational level at t1. A variable indicating the highest parental educational level in four categories was constructed: 1) upper secondary school ≤2 years or less; 2) upper secondary school ≥3 years; 3) tertiary education ≤2 years; and 4) tertiary education ≥3 years.

*Parental country of birth* was based on register information about the father's and the mother's country of birth. A variable was constructed distinguishing between 1) participants with at least one parent born in Sweden; and 2) those with two parents born outside Sweden.

### 2.3 Analytical strategy and statistical methods

First, the prevalence of psychosomatic complaints and gender differences were examined by descriptive statistics and chi-square tests. To assess the links between the frequency, number, and persistence of psychosomatic complaints in adolescence (at t1 and t2) and depression and anxiety symptoms in young adulthood (at t3), we examined the proportions of adolescents with psychosomatic complaints at t1 and/or at t2 who reported depression and anxiety symptoms at t3, and performed multivariable binary logistic regression analyses (stratified by gender), regressing depression and anxiety symptoms on psychosomatic complaints. In the analyses of the frequency and number of complaints, we showed different models for each time point: firstly, the association between psychosomatic complaints at t1 and depression and anxiety symptoms at t3 (disregarding psychosomatic complaints at t2), and secondly, the association between psychosomatic complaints at t2 and depression and anxiety symptoms at t3 (disregarding psychosomatic complaints at t1). For the analyses of the persistence of psychosomatic complaints, the independent variable was constructed based on self-reports on psychosomatic complaints from both t1 and t2 (with the four following categories: psychosomatic complaints neither at t1 nor at t2, at t1 but not at t2, at t2 but not at t1, and both at t1 and t2). We also performed analyses of the total sample and included interaction terms between psychosomatic complaints and gender, which were evaluated with Wald tests. All models were adjusted for family type, parental education, and parental country of birth (the crude models, without sociodemographic covariates, are demonstrated in the Appendix, Table A.4). To take the clustered nature of the data into account, with students nested in classes at t1, we estimated robust standard errors, clustering by school class. The number of classes was 335. In addition to odds ratios (OR) that were reported along with 95% confidence intervals (CI) for each regression model, we also estimated average marginal effects (AME) (presented in the Appendix, Table A.5). Furthermore, we performed analyses using continuous scales of depression and anxiety symptoms (presented in the Appendix, Table A.6). All statistical analyses were performed using Stata 16.0 (StataCorp, 2019).

### 2.4 Ethics

Ethical approval for this study was obtained from the Swedish Ethical Review Authority (ref. 2021-06504-01; 2022-02781-02; 2022-06502-02). Informed consent was obtained from all participants in written form.

## 3 Results

The frequency, type, number, and persistence of psychosomatic complaints at t1 and t2 and depression and anxiety symptoms at t3, for all and separately by gender, are presented in Table 1. The results show that psychosomatic complaints were significantly more common in girls than in boys across all operationalisations. As to the frequency of complaints, more girls than boys reported at least one complaint “More often than weekly” or “Daily” at both t1 and t2. In both genders, the most common type of psychosomatic complaint of those under study was difficulties falling asleep, and stomach ache was the least frequent. Girls tended to experience higher numbers of complaints than boys at both t1 and t2. Psychosomatic complaints were also more persistent in girls. Although there was a higher prevalence of depression symptoms in females (27.1%) than in males (24.0%), the difference by gender was not statistically significant (p = 0.070). Anxiety symptoms were more common in females (34.4%) than in males (17.5%), the difference being statistically significant (p < 0.001).

**Table 1:** Descriptives of psychosomatic complaints (frequency, type, number and persistence of complaints) at t1 and t2 and of depression and anxiety symptoms at t3, in the total sample and stratified by gender. Differences by gender assessed with χ^2^ tests.

	All (n = 2779)		Males (n = 1169)		Females (n = 1610)		p
	n	%	n	%	n	%	
*Frequency of complaints*							
At least one complaint (t1)							
Less often than weekly	1019	36.6	555	47.5	464	28.8	<0.001
Weekly	538	19.4	240	20.5	298	18.5	
More often than weekly	856	30.8	277	23.7	579	36.0	
Daily	366	13.2	97	8.3	269	16.7	
At least one complaint (t2)							
Less often than weekly	885	31.8	480	41.0	405	25.2	<0.001
Weekly	596	21.4	272	23.3	324	20.1	
More often than weekly	910	32.8	314	26.9	596	37.0	
Daily	388	14.0	103	8.8	285	17.7	
*Type of complaints*							
More often than weekly/daily (t1)							
Stomach ache	397	14.3	79	6.8	318	19.8	<0.001
Headache	628	22.6	129	11.0	499	31.0	<0.001
Difficulties falling asleep	819	29.5	283	24.2	536	33.3	<0.001
More often than weekly/daily (t2)							
Stomach ache	461	16.6	103	8.8	358	22.2	<0.001
Headache	634	22.8	116	9.9	518	32.2	<0.001
Difficulties falling asleep	857	30.8	329	28.1	528	32.8	0.009
*Number of complaints*							
Number of complaints more often than weekly/daily (t1)							
0 complaint	1557	56.0	795	68.0	762	47.3	
1 complaint	739	26.6	273	23.4	466	28.9	
≥2 complaints	483	17.4	101	8.6	382	23.7	<0.001
Number of complaints more often than weekly/daily (t2)							
0 complaint	1481	53.3	752	64.3	729	45.3	
1 complaint	785	28.3	306	26.2	479	29.8	
≥2 complaints	513	18.5	111	9.5	402	25.0	<0.001
*Persistence of complaints*							
≥2 complaints more often than weekly/daily							
Neither at t1 nor at t2	2028	73.0	989	84.6	1039	64.5	<0.001
At t1 but not at t2	238	8.6	69	5.9	169	10.5	
At t2 but not at t1	268	9.6	79	6.8	189	11.8	
At t1 and t2	245	8.8	32	2.7	213	13.2	
*Depression and anxiety symptoms*							
Depression symptoms (t3)	717	25.8	281	24.0	436	27.1	0.070
Anxiety symptoms (t3)	759	27.3	205	17.5	554	34.4	<0.001

The frequency, type and number of psychosomatic complaints based on the full baseline sample are presented in the Appendix, Table A.1. Comparison of the percentages in Table 1 and Table A.1 indicates that there was no systematic bias in the attrition with regards to psychosomatic complaints at t1. Descriptives of depression and anxiety symptoms (for the separate items and the full scales) and distributions of the covariates, for all and stratified by gender, are shown in the Appendix, Table A.2.

Next, we examined the links between the frequency, number, and persistence of psychosomatic complaints at t1 and t2 and depression and anxiety symptoms at t3, with results displayed in Table 2, Table 3, Table 4.

**Table 2:** Per cent and odds ratios (OR) with 95% confidence intervals (CI) from binary logistic regression models analysing the associations between the frequency of psychosomatic complaints at t1 (upper part of the table) and t2 (lower part of the table) and depression and anxiety symptoms at t3, stratified by gender. Models adjust for family type, parental education, and parental country of birth.

	Depression symptoms (t3)						Anxiety symptoms (t3)					
	Males (n = 1169)			Females (n = 1610)			Males (n = 1169)			Females (n = 1610)		
	%	OR	95% CI	%	OR	95% CI	%	OR	95% CI	%	OR	95% CI
Frequency of complaints^a^ (t1)												
Less often than weekly (ref.)	18.4	1.00	–	20.3	1.00	–	12.6	1.00	–	24.4	1.00	–
Weekly	23.8	1.47*	1.01; 2.16	23.2	1.20	0.84; 1.70	17.1	1.47	0.98; 2.21	28.2	1.22	0.86; 1.74
More often than weekly	32.1	2.11***	1.50; 2.98	27.8	1.47*	1.08; 2.01	23.8	2.13***	1.44; 3.15	37.0	1.79***	1.34; 2.39
Daily	34.0	2.12**	1.31; 3.45	41.6	2.73***	1.94; 3.84	28.9	2.64***	1.60; 4.37	53.2	3.45***	2.46; 4.85
Frequency of complaints^a^ (t2)												
Less often than weekly (ref.)	16.0	1.00	–	19.0	1.00	–	11.0	1.00	–	19.5	1.00	–
Weekly	23.9	1.70*	1.13; 2.56	19.1	0.99	0.72; 1.31	18.4	1.84**	1.21; 2.77	32.1	1.92***	1.39; 2.65
More often than weekly	30.3	2.27***	1.61; 3.19	29.4	1.71**	1.26; 2.33	20.7	2.09***	1.41; 3.08	36.4	2.30***	1.71; 3.08
Daily	42.7	3.78***	2.31; 6.19	42.8	*3*.03***	2.14; 4.30	35.9	4.43***	2.77; 7.08	54.0	4.70***	3.33; 6.63

^a^ At least one psychosomatic complaint (stomach ache, headache and/or difficulties falling asleep). Figures in italics indicate statistically significant difference from the category “Weekly” (p < 0.05). ***p < 0.001 **p < 0.01 *p < 0.05.

**Table 3:** Per cent and odds ratios (OR) with 95% confidence intervals (CI) from binary logistic regression models analysing the associations between the number of psychosomatic complaints at t1 (upper part of table) and at t2 (lower part of table) and depression and anxiety symptoms at t3, stratified by gender. Models adjust for family type, parental education, and parental country of birth.

	Depression symptoms (t3)						Anxiety symptoms (t3)					
	Males (n = 1169)			Females (n = 1610)			Males (n = 1169)			Females (n = 1610)		
	%	OR	95% CI	%	OR	95% CI	%	OR	95% CI	%	OR	95% CI
Number of complaints^a^ (t1)												
0 complaint (ref.)	20.0	1.00	–	21.4	1.00	–	14.0	1.00	–	25.9	1.00	–
1 complaint	31.1	1.76**	1.28; 2.40	27.7	1.37*	1.04; 1.81	23.8	1.86***	1.31; 2.64	36.1	1.59***	1.24; 2.05
≥2 complaints	36.6	2.22**	1.42; 3.46	37.7	2.14***	1.61; 2.85	28.7	2.40***	1.49; 3.86	49.5	2.74***	2.09; 3.60
Number of complaints^a^ (t2)												
0 complaint (ref.)	18.9	1.00	–	19.1	1.00	–	13.7	1.00	–	25.1	1.00	–
1 complaint	30.7	1.88***	1.40; 2.54	28.6	1.68**	1.25; 2.27	20.9	1.64**	1.16; 2.31	35.3	1.60***	1.26; 2.03
≥2 complaints	40.5	2.77***	1.79; 4.27	39.8	2.67***	2.02; 3.53	34.2	3.20***	2.11; 4.85	50.3	2.93***	2.23; 3.84

^a^ Number of complaints more often than weekly (includes the “More often than weekly” and “Daily” categories). Figures in italics indicate statistically significant difference from the category “≥2 complaints” (p < 0.05). ***p < 0.001 **p < 0.01 *p < 0.05.

**Table 4:** Per cent and odds ratios (OR) with 95% confidence intervals (CI) from binary logistic regression models analysing the associations between the persistence of psychosomatic complaints from t1 to t2 and depression and anxiety symptoms at t3, stratified by gender. Models adjust for family type, parental education, and parental country of birth.

	Depression symptoms (t3)						Anxiety symptoms (t3)					
	Males (n = 1169)			Females (n = 1610)			Males (n = 1169)			Females (n = 1610)		
	%	OR	95% CI	%	OR	95% CI	%	OR	95% CI	%	OR	95% CI
Persistence of complaints^a^												
Neither at t1 nor at t2 (ref.)	21.2	1.00	–	21.7	1.00	–	15.0	1.00	–	27.1	1.00	–
At t1 but not at t2	37.7	2.13**	1.29; 3.53	30.2	1.51*	1.03; 2.21	27.5	2.05*	1.18; 3.57	42.0	1.93***	1.36; 2.74
At t2 but not at t1	43.0	2.59***	1.65; 4.06	35.5	1.85***	1.32; 2.59	35.4	2.98***	1.88; 4.73	44.4	*2*.12***	1.53; 2.93
At t1 and t2	34.4	1.92	0.91; 4.07	43.7	2.71***	1.98; 3.70	31.3	2.67*	1.25; 5.68	55.4	3.27***	2.37; 4.51

^a^ ≥2 psychosomatic complaints more often than weekly (includes the “More often than weekly” and “Daily” categories). Figures in italics indicate statistically significant difference from the category “At t1 and t2” (p < 0.05). ***p < 0.001 **p < 0.01 *p < 0.05.

Table 2 shows the results of the analyses of the frequency of psychosomatic complaints and subsequent depression and anxiety symptoms. Examining the proportions of individuals reporting depression and anxiety symptoms at t3 by the frequency of psychosomatic complaints (at least one type) at t1 (upper part of the table) and at t2 (lower part of the table), the percentages reveal clear gradients. The more frequent psychosomatic complaints at t1 and t2, the more common were depression and anxiety symptoms at t3. The binary logistic regression analyses confirmed this descriptive pattern. The more frequent psychosomatic complaints at t1 and at t2, the higher was the likelihood of reporting depression and anxiety symptoms at t3, in a graded manner. In all the cases, the highest odds ratio was seen for the “More often than weekly” and “Daily” categories, with daily psychosomatic complaints being most strongly associated with subsequent depressive and anxiety symptoms. Overall, the same pattern was seen for depression and anxiety symptoms (with slightly higher odds ratios for the latter) and for males and females alike. Analyses of the total sample including interaction terms between the frequency of psychosomatic complaints and gender were not statistically significant (not presented in Table).

While the analyses presented in Table 2 show the results for at least one psychosomatic complaint but without distinguishing between different type of complaints, the same set of analyses for each psychosomatic complaint are presented in the Appendix, Table A.3. These results largely reflect those of the analyses from Table 2, showing that more frequent psychosomatic complaints were more strongly associated with subsequent depression and anxiety symptoms. The associations pointed in the same direction for all complaints indicating that the results presented in Table 2 were not driven by only one specific type of complaint. Analyses of the total sample testing for interactions between each type of complaint and gender (not presented in Table) did not reveal any statistically significant interaction terms.

Table 3 presents the links between depression and anxiety symptoms at t3 and the number of psychosomatic complaints at t1 and at t2 (reported more often than weekly, i.e., including the categories “More often than weekly” and “Daily”). As in the analysis with the frequency of psychosomatic complaints, the proportions of individuals reporting depression and anxiety symptoms at t3 by the number of psychosomatic complaints at t1 and at t2 showed clear gradients: The proportions with depression and anxiety symptoms were higher among adolescents who also reported a higher number of psychosomatic complaints. In a consistent manner, the highest odds ratios were seen for the category “≥2 complaints”. Having only one complaint more than weekly was also associated with depression and anxiety symptoms in all the cases. In a majority of the cases (mostly in females), the estimates for the category “≥2 complaints” differed significantly from those for the category “1 complaint” (shown in italics). Analyses of the total sample did not show any statistically significant interactions between the number of psychosomatic complaints and gender (not presented in Table).

The associations between the persistence of psychosomatic complaints and subsequent depression and anxiety symptoms are displayed in Table 4. Psychosomatic complaints were in this analysis defined as two or more psychosomatic complaints more than weekly (i.e., including the “More often than weekly” and “Daily” categories). In general, adolescents who reported psychosomatic complaints at t1 and/or at t2 showed higher proportions of depression and anxiety symptoms at t3, compared with those who did not report psychosomatic complaints neither at t1 nor at t2. In the binary logistic regression analyses, higher odds ratios of having depression and anxiety symptoms were generally observed for more recent than earlier complaints, but especially for females, the differences were relatively small. Among females, the highest odds ratio for both depression and anxiety symptoms was found among those reporting persistent psychosomatic complaints. This pattern was not seen among males. It should be noted, however, that only a small number of boys reported psychosomatic complaints at both t1 and t2, which might have affected the results concerning the persistence of complaints in males. Analyses of the total sample did not show any statistically significant interactions between the persistence of psychosomatic complaints and gender.

Although all the models in Table 2, Table 3, Table 4 adjusted for family type, parental education, and parental country of birth, the corresponding analyses without any controls showed very similar estimates (see the Appendix, Table A.4). The patterns were also similar when average marginal effects were reported (Table A.5) and when linear regression analyses were performed using the full continuous scales of depression and anxiety symptoms (Table A.6).

## 4 Discussion

This study aimed to examine the longitudinal associations between the frequency, number, and persistence of adolescent psychosomatic complaints and depression and anxiety symptoms in young adulthood. The descriptive and multivariable binary logistic regression analyses showed that psychosomatic complaints in adolescence were clearly and consistently associated with an increased likelihood of subsequent depression and anxiety symptoms. Our findings add to those from earlier studies showing that psychosomatic complaints in youth predicted subsequent adverse mental health conditions (Bohman et al., 2012, 2018; Giannotta et al., 2022; Shanahan et al., 2015; Shelby et al., 2013) by demonstrating that the frequency, number, and persistence of complaints were all associated with later depressive and anxiety symptoms.

Regarding both the frequency and the number of psychosomatic complaints in adolescence, our analyses presented graded associations with depression and anxiety symptoms in young adulthood. These results align with the findings of Högberg et al. (2023), who showed that there was no bimodal distribution of adolescent psychosomatic complaints distinguishing between ‘trivial’ and ‘more severe’ levels of complaints, but that psychosomatic complaints rather present at a continuum with no distinct clusters. As one of very few studies investigating prospective links between repeated measurements of psychosomatic complaints and subsequent symptoms of depression and anxiety, the present study also indicated that persistent psychosomatic complaints (measured at both time points) displayed a stronger association with later depression and anxiety symptoms in females. Furthermore, recent psychosomatic complaints were more strongly associated with depression and anxiety symptoms in males (only a few of whom had persistent psychosomatic complaints). In general, our result for persistence reflects the findings of Shanahan et al. (2015), who reported that continuous somatic complaints at ages 9–16 years predicted the highest risk of mental health conditions in young adulthood. Our findings, along with those of Shanahan et al. (2015), indicate that measurements of psychosomatic complaints from several time-points contribute to identifying individuals of particularly high risk of developing mental health problems, and especially so for girls. Repeated measurements not only allow to capture the persistence of such complaints, but also to measure more recent psychosomatic complaints. Moreover, the co-occurrence of depression, anxiety and psychosomatic symptoms has been shown to be high and stable from adolescence to midlife (Lallukka et al., 2019) which, along with the findings of the current study, highlights the importance of detection of psychosomatic complaints in the earlier phase.

In the present study, a female excess in psychosomatic complaints was observed, which aligns with prior research (Aanesen et al., 2017; Högberg et al., 2020; Inchley, 2020; Ravens-Sieberer et al., 2009; Sweeting et al., 2007; Torsheim et al., 2006); however, the associations between psychosomatic complaints and subsequent symptoms of depression and anxiety were similar in both genders (with some exceptions), also reflecting previous inquiry (e.g., Giannotta et al., 2022). The results also showed that while anxiety symptoms were much more common in females than in males, the female excess in depression symptoms was minor and not statistically significant. The latter finding was unexpected and in contrast to previous research (Salk et al., 2017). However, when the full t3 sample was used (i.e. only excluding cases with missing information on PHQ-2, n = 3314), the proportions of males and females reporting depression symptoms were similar to those in the study sample, but a statistically significant gender difference in depression symptoms was observed (males: 23.7%; females 27.3%, p = 0.017) (not presented in Table). Furthermore, the descriptives of the separate items (presented in the Appendix, Table A2) indicated that there was no gender difference in the symptom “Little interest or pleasure in doing things” (p = 0.415), while the distribution of the symptom “Feeling down, depressed, or hopeless” did differ by gender, with a higher prevalence among females (p < 0.001). Also, the mean of the depression symptoms scale was higher in females than in males (p = 0.002).

Given the high prevalence of psychosomatic complaints in adolescence, particularly sleeping problems, which are frequently comorbid with both depression and anxiety (Buysse et al., 2008; Chorney et al., 2008; Orchard et al., 2020; Spoormaker & Van den Bout, 2005), the assessment of such complaints and corresponding interventions among adolescents appear particularly important. Although sleeping problems are considered a symptom of depression and anxiety disorders (American Psychiatric Association, 2022; World Health Organization, 2019), recent evidence suggests that they could occur earlier than a diagnosis is made (e.g., Gradisar et al., 2022), and therefore earlier evaluation of these problems is also necessary. To summarise, the assessment of psychosomatic complaints is important during adolescence when frequent, higher in number, and persistent psychosomatic complaints could predict subsequent mental health problems, including depression and anxiety symptoms.

### 4.1 Strengths and limitations

This study was based on longitudinal data containing three waves, which is a merit, since it allowed us to examine psychosomatic complaints over a two-year period, as well as the links with subsequent depression and anxiety symptoms. However, there are also limitations. While the data was based on a nationally representative sample of adolescents, the attrition in several steps may have compromised the representativeness. Notwithstanding, comparison between the full t1 sample and the three waves study sample indicated that there was no systematic bias in the attrition with regards to psychosomatic complaints at t1. Since depression and anxiety symptoms were only measured at t3, it cannot be ruled out that part of the reported associations in fact reflect continuous problems of depression or anxiety from adolescence into young adulthood. Additionally, we could not exclude that some experienced psychosomatic complaints were indicators of existing physical illnesses (e.g., eyesight problems, sinus, dental or jaw problems, infections, gastroesophageal reflux and others). Furthermore, although three of the most common and often co-occurring (Luntamo et al., 2012) psychosomatic complaints were measured in the current study, future research should include a broader list of psychosomatic complaints, since some prior studies showed that the associations between various psychosomatic complaints and mental health problems may differ by types of complaints (e.g., Egger et al., 1999; Giannotta et al., 2022). It should also be noted that the measures of psychosomatic complaints and depression and anxiety symptoms were based on adolescents’ self-reports and hence may be subject to, e.g., differences in interpretation, or social desirability bias. Finally, it should be acknowledged that since the data was collected in Sweden, a country which has comparatively high levels of adolescent psychosomatic complaints (Hagquist et al., 2019; Potrebny et al., 2017), the scope of generalisability to other national and geographical contexts may be limited. To corroborate the findings of the current study, there is a need for further research based on data from other settings.

## 5 Conclusions

The current study showed that self-reported psychosomatic complaints in adolescence were associated with depression and anxiety symptoms in young adulthood. This was true across the frequency, number, and persistence of psychosomatic complaints. Regarding the analyses of persistence, a general finding was that adolescent girls who reported persistent psychosomatic complaints had the highest likelihood of reporting subsequent depression and anxiety symptoms. However, even those adolescents who experienced psychosomatic complaints at one time point only (particularly recent psychosomatic complaints) had an increased likelihood of experiencing depression and anxiety symptoms in young adulthood. Taken together, the results indicate that adolescent psychosomatic complaints should not be disregarded as trivial. Overall, self-reported psychosomatic complaints could be a useful identifier for adolescents at risk of developing future depression and anxiety, and therefore targeting psychosomatic complaints in adolescence is important and may have long-term mental health benefits. Furthermore, repeated measurements of psychosomatic complaints may help to identify the most vulnerable group. The findings also suggest that both assessment and treatment of depression and anxiety symptoms should address psychosomatic complaints.

## Ethical statement

The study was approved by the Swedish Ethical Review Authority (ref. 2021-06504-01; 2022-02781-02; 2022-06502-02). According to the Swedish Ethical Review Act (SFS 2003:460), parental consent is not required for adolescents aged 15 or older if they realise what the research means for them. Informed consent was obtained from all study participants. All methods were carried out in accordance with relevant guidelines and regulations.

## Authors’ contributions

Karina Grigorian, Sara Brolin Låftman, Viveca Östberg and Jonas Raninen contributed to the conceptualisation and the design of the study. The Futura01 data were collected in a research project headed by Jonas Raninen. Karina Grigorian performed the statistical analyses and drafted the manuscript. Sara Brolin Låftman, Viveca Östberg, Jonas Raninen and Johan Åhlén critically revised drafts of the manuscript. All authors read and approved the final manuscript.

## Availability of data and materials

The data that support the findings of this study are available from 10.13039/501100004047Karolinska Institutet but restrictions apply to the availability of these data, which were used under license for the current study, and so are not publicly available. Data are however available from the authors upon reasonable request and with permission of Karolinska Institutet and ethical approval from the Swedish Ethical Review Authority.

## Declaration of competing interest

None.

## Data Availability

The authors do not have permission to share data.
