# Supplementary material for: Prospective associations between psychosomatic complaints in adolescence and depression and anxiety symptoms in young adulthood: A Swedish national cohort study
Source: SSM Popul Health. 2023 Sep 4;24:101509. doi: 10.1016/j.ssmph.2023.101509 (PMC10500464; doi:10.1016/j.ssmph.2023.101509)
Supplement: Multimedia component 1 [file mmc1.docx]

**Appendix**

**Table A.1** Descriptives of psychosomatic complaints (frequency, type, and number of complaints) in the total sample and stratified by gender. Differences by gender assessed with χ^2^ tests. Full sample at t1 (n=5263).

|  | All  (n=5263) | | Males  (n=2600) | | Females  (n=2663) | | p |
| --- | --- | --- | --- | --- | --- | --- | --- |
|  | n | % | n | % | n | % |  |
| *Frequency of complaints* |  |  |  |  |  |  |  |
| At least one complaint (t1) |  |  |  |  |  |  |  |
| Less often than weekly | 1904 | 36.2 | 1179 | 45.4 | 725 | 27.2 | <0.001 |
| Weekly | 1030 | 19.6 | 536 | 20.6 | 494 | 18.5 |  |
| More often than weekly | 1611 | 30.6 | 643 | 24.7 | 968 | 36.4 |  |
| Daily | 718 | 13.6 | 242 | 9.3 | 476 | 17.9 |  |
|  |  |  |  |  |  |  |  |
| *Type of complaints* |  |  |  |  |  |  |  |
| More often than weekly/daily (t1) |  |  |  |  |  |  |  |
| Stomach ache | 745 | 14.2 | 197 | 7.6 | 548 | 20.6 | <0.001 |
| Headache | 1186 | 22.5 | 336 | 12.9 | 850 | 31.9 | <0.001 |
| Difficulties falling asleep | 1588 | 30.2 | 666 | 25.6 | 922 | 34.6 | <0.001 |
|  |  |  |  |  |  |  |  |
| *Number of complaints* |  |  |  |  |  |  |  |
| Number of complaints more often than weekly/daily (t1) |  |  |  |  |  |  |  |
| 0 complaint | 2934 | 55.7 | 1715 | 66.0 | 1219 | 45.8 | <0.001 |
| 1 complaint | 1404 | 26.7 | 622 | 23.9 | 782 | 29.4 |  |
| ≥ 2 complaints | 925 | 17.6 | 263 | 10.1 | 662 | 24.8 |  |

**Table A.2** Descriptives of depression and anxiety symptoms (dichotomous measures, separate items, and full continuous scales) and of covariates, and differences by gender assessed with χ^2^ and unpaired t tests (n=2779).

|  | All  (n=2779) | | Males  (n=1169) | | Females  (n=1610) | | p |
| --- | --- | --- | --- | --- | --- | --- | --- |
|  | n | % | n | % | n | % |  |
|  |  |  |  |  |  |  |  |
| Depression symptoms (t3) | 717 | 25.8 | 281 | 24.0 | 436 | 27.1 | 0.070 |
| Little interest or pleasure in doing things |  |  |  |  |  |  |  |
| Not at all | 1095 | 39.4 | 469 | 40.1 | 626 | 38.8 |  |
| Several days | 1066 | 38.4 | 434 | 37.1 | 632 | 39.2 |  |
| More than half days | 382 | 13.7 | 157 | 13.5 | 225 | 14.0 |  |
| Nearly every day | 236 | 8.5 | 109 | 9.3 | 127 | 8.0 | 0.415 |
| Feeling down, depressed, or hopeless |  |  |  |  |  |  |  |
| Not at all | 1256 | 45.2 | 615 | 52.6 | 641 | 39.8 |  |
| Several days | 1023 | 36.8 | 380 | 32.5 | 643 | 39.9 |  |
| More than half days | 286 | 10.3 | 96 | 8.2 | 190 | 11.8 |  |
| Nearly every day | 214 | 7.7 | 78 | 6.7 | 136 | 8.5 | <0.001 |
|  |  |  |  |  |  |  |  |
|  | M | S.D. | M | S.D. | M | S.D. |  |
| Depression symptoms scale (0 – 6) | 1.72 | 1.59 | 1.61 | 1.58 | 1.80 | 1.60 | 0.002 |
|  |  |  |  |  |  |  |  |
| Anxiety symptoms (t3) | 759 | 27.3 | 205 | 17.5 | 554 | 34.4 | <0.001 |
| Feeling nervous, anxious or on edge |  |  |  |  |  |  |  |
| Not at all | 801 | 28.8 | 484 | 41.4 | 317 | 19.7 |  |
| Several days | 1271 | 45.8 | 501 | 42.9 | 770 | 47.8 |  |
| More than half days | 340 | 12.2 | 96 | 8.2 | 244 | 15.2 |  |
| Nearly every day | 367 | 13.2 | 88 | 7.5 | 279 | 17.3 | <0.001 |
| Not being able to stop or control worrying |  |  |  |  |  |  |  |
| Not at all | 1302 | 46.8 | 719 | 61.5 | 583 | 36.2 |  |
| Several days | 952 | 34.3 | 305 | 26.1 | 647 | 40.2 |  |
| More than half days | 275 | 9.9 | 83 | 7.1 | 192 | 11.9 |  |
| Nearly every day | 250 | 9.0 | 62 | 5.3 | 188 | 11.7 | <0.001 |
|  |  |  |  |  |  |  |  |
|  | M | S.D. | M | S.D. | M | S.D. |  |
| Anxiety symptoms scale (0 – 6) | 1.91 | 1.78 | 1.38 | 1.58 | 2.29 | 1.82 | <0.001 |
|  |  |  |  |  |  |  |  |
|  | n | % | n | % | n | % |  |
| Family type (t1) |  |  |  |  |  |  |  |
| Both parents | 1989 | 71.6 | 863 | 73.8 | 1126 | 70.0 | 0.018 |
| Shared residence | 367 | 13.2 | 157 | 13.4 | 210 | 13.0 |  |
| Single parent | 359 | 12.9 | 124 | 10.6 | 235 | 14.6 |  |
| Other | 64 | 2.3 | 25 | 2.2 | 39 | 2.4 |  |
| Parental education |  |  |  |  |  |  |  |
| Upper secondary school (≤2 years) or less | 405 | 14.6 | 149 | 12.7 | 256 | 15.9 | 0.059 |
| Upper secondary school (≥3 years) | 521 | 18.7 | 214 | 18.3 | 307 | 19.1 |  |
| Tertiary education (≤2 years) | 519 | 18.7 | 216 | 18.5 | 303 | 18.8 |  |
| Tertiary education (≥3 years) | 1334 | 48.0 | 590 | 50.5 | 744 | 46.2 |  |
| Parental country of birth |  |  |  |  |  |  |  |
| At least one parent born in Sweden | 2356 | 84.8 | 1000 | 85.5 | 1356 | 84.2 | 0.339 |
| Both parents born outside Sweden | 423 | 15.2 | 169 | 14.5 | 254 | 15.8 |  |

**Table A.3** Per cent and odds ratios (OR) with 95% confidence intervals (CI) from binary logistic regression models analysing the associations between the type and frequency of psychosomatic complaints at t1 (upper part of table) and at t2 (lower part of table) and depression and anxiety symptoms at t3, stratified by gender. Models mutually adjust for all three psychosomatic complaints, family type, parental education, and parental country of birth.

|  | Depression symptoms (t3) | | | | | | Anxiety symptoms (t3) | | | | | |
| --- | --- | --- | --- | --- | --- | --- | --- | --- | --- | --- | --- | --- |
|  |  |  |  |  |  |  |  |  |  |  |  |  |
|  | Males (n=1169) | | | Females (n=1610) | | | Males (n=1169) | | | Females (n=1610) | | |
|  |  |  |  |  |  |  |  |  |  |  |  |  |
|  | % | OR | 95% CI | % | OR | 95% CI | % | OR | 95% CI | % | OR | 95% CI |
| Stomach ache (t1) |  |  |  |  |  |  |  |  |  |  |  |  |
| Less often than weekly (ref.) | 21.3 | 1.00 | - | 24.6 | 1.00 | - | 16.1 | 1.00 | - | 30.1 | 1.00 | - |
| Weekly | 34.2 | 1.66* | 1.10; 2.51 | 25.4 | 0.89 | 0.63; 1.25 | 22.8 | 1.29 | 0.77; 2.17 | 35.9 | 1.06 | 0.80; 1.42 |
| More often than weekly^a^ | 43.0 | 2.25** | 1.32; 3.82 | 36.5 | 1.39* | 1.03; 1.87 | 27.9 | 1.52 | 0.89; 2.60 | 47.2 | 1.54** | 1.17; 2.03 |
| Headache (t1) |  |  |  |  |  |  |  |  |  |  |  |  |
| Less often than weekly (ref.) | 21.3 | 1.00 | - | 24.3 | 1.00 | - | 15.9 | 1.00 | - | 29.4 | 1.00 | - |
| Weekly | 29.7 | 1.40 | 0.94; 2.09 | 25.5 | 0.93 | 0.68; 1.26 | 18.9 | 1.05 | 0.64; 1.72 | 35.8 | 1.09 | 0.80; 1.48 |
| More often than weekly^a^ | 36.4 | 1.49 | 0.97; 2.30 | 32.7 | 1.10 | 0.83; 1.46 | 27.1 | 1.37 | 0.86; 2.17 | 41.9 | 1.20 | 0.92; 1.56 |
| Difficulties falling asleep (t1) |  |  |  |  |  |  |  |  |  |  |  |  |
| Less often than weekly (ref.) | 21.1 | 1.00 | - | 21.5 | 1.00 | - | 13.6 | 1.00 | - | 26.8 | 1.00 | - |
| Weekly | 25.2 | 1.15 | 0.78; 1.69 | 28.4 | 1.41 | 0.99; 2.00 | 21.6 | 1.68* | 1.12; 2.53 | 34.7 | 1.36 | 0.99; 1.87 |
| More often than weekly^a^ | 30.7 | 1.28 | 0.89; 1.84 | 35.1 | 1.77*** | 1.34; 2.35 | 25.1 | 1.81** | 1.26; 2.60 | 45.9 | 1.94*** | 1.48; 2.54 |
|  |  |  |  |  |  |  |  |  |  |  |  |  |
| Stomach ache (t2) |  |  |  |  |  |  |  |  |  |  |  |  |
| Less often than weekly (ref.) | 22.4 | 1.00 | - | 24.9 | 1.00 | - | 16.1 | 1.00 | - | 28.8 | 1.00 | - |
| Weekly | 25.0 | 0.97 | 0.61; 1.55 | 23.1 | 0.69* | 0.49; 0.97 | 20.2 | 1.15 | 0.69; 1.92 | 36.6 | 1.12 | 0.84; 1.49 |
| More often than weekly^a^ | 38.8 | 1.65* | 1.03; 2.63 | 36.0 | 1.18 | 0.91; 1.54 | 29.1 | 1.59 | 0.93; 2.72 | 48.3 | 1.68*** | 1.26; 2.22 |
| Headache (t2) |  |  |  |  |  |  |  |  |  |  |  |  |
| Less often than weekly (ref.) | 20.3 | 1.00 | - | 22.3 | 1.00 | - | 14.9 | 1.00 | - | 27.3 | 1.00 | - |
| Weekly | 33.1 | 1.63* | 1.10; 2.40 | 27.5 | 1.08 | 0.80; 1.48 | 20.9 | 1.26 | 0.78; 2.03 | 35.8 | 1.12 | 0.83; 1.52 |
| More often than weekly^a^ | 38.8 | 1.72* | 1.11; 2.67 | 34.0 | 1.34* | 1.04; 1.73 | 32.8 | 2.07** | 1.26; 3.40 | 44.2 | 1.44* | 1.09; 1.90 |
| Difficulties falling asleep (t2) |  |  |  |  |  |  |  |  |  |  |  |  |
| Less often than weekly (ref.) | 18.7 | 1.00 | - | 19.2 | 1.00 | - | 13.9 | 1.00 | - | 23.9 | 1.00 | - |
| Weekly | 26.4 | 1.45 | 0.97; 2.17 | 26.7 | 1.48* | 1.06; 2.06 | 18.8 | 1.31 | 0.85; 2.00 | 41.8 | 2.09*** | 1.55; 2.83 |
| More often than weekly^a^ | 32.8 | 1.74*** | 1.28; 2.36 | 38.8 | 2.31*** | 1.77; 3.03 | 23.7 | 1.53* | 1.07; 2.19 | 45.5 | 2.06*** | 1.55; 2.73 |

^a^ Includes the “More often than weekly” and “Daily” categories.

***p<0.001 **p<0.01 *p<0.05

**Table A.4** Odds ratios (OR) with 95% confidence intervals (CI) from binary logistic regression models analysing the associations between the frequency, number, and persistence of psychosomatic complaints at t1 and t2 and depression and anxiety symptoms at t3, stratified by gender (crude logistic regression analyses, without any controls).

|  | Depression symptoms (t3) | | | | Anxiety symptoms (t3) | | | |
| --- | --- | --- | --- | --- | --- | --- | --- | --- |
|  | Males (n=1169) | | Females (n=1610) | | Males (n=1169) | | Females (n=1610) | |
|  | OR | 95% CI | OR | 95% CI | OR | 95% CI | OR | 95% CI |
| Frequency of complaints^a^ (t1) |  |  |  |  |  |  |  |  |
| Less often than weekly (ref.) | 1.00 | - | 1.00 | - | 1.00 | - | 1.00 | - |
| Weekly | 1.38 | 0.95; 2.01 | 1.19 | 0.84; 1.68 | 1.43 | 0.95; 2.15 | 1.22 | 0.86; 1.73 |
| More often than weekly | 2.10*** | 1.51; 2.92 | 1.52** | 1.11; 2.06 | 2.17*** | 1.48; 3.18 | *1.82**** | 1.36; 2.43 |
| Daily | 2.29*** | 1.45; 3.61 | *2.81**** | 2.00; 3.94 | *2.81**** | 1.73; 4.58 | *3.53**** | 2.51; 4.94 |
|  |  |  |  |  |  |  |  |  |
| Frequency of complaints^a^ (t2) |  |  |  |  |  |  |  |  |
| Less often than weekly (ref.) | 1.00 | - | 1.00 | - | 1.00 | - | 1.00 | - |
| Weekly | 1.64* | 1.10; 2.45 | 1.01 | 0.68; 1.49 | 1.81** | 1.21; 2.73 | 1.95*** | 1.42; 2.68 |
| More often than weekly | 2.27*** | 1.63; 3.16 | *1.77*** | 1.30; 2.40 | 2.10*** | 1.43; 3.09 | 2.36*** | 1.76; 3.18 |
| Daily | *3.90**** | 2.45; 6.23 | *3.19**** | 2.26; 4.50 | *4.52**** | 2.84; 7.19 | *4.85**** | 3.43; 6.86 |
|  |  |  |  |  |  |  |  |  |
| Number of complaints^b^ (t1) |  |  |  |  |  |  |  |  |
| 0 complaint (ref.) | 1.00 | - | 1.00 | - | 1.00 | - | 1.00 | - |
| 1 complaint | 1.81*** | 1.34; 2.45 | *1.41** | 1.07; 1.85 | 1.93*** | 1.37; 2.71 | *1.62**** | 1.26; 2.08 |
| ≥2 complaints | 2.31*** | 1.50; 3.57 | 2.22*** | 1.68; 2.94 | 2.48*** | 1.57; 3.93 | 2.81*** | 2.14; 3.68 |
|  |  |  |  |  |  |  |  |  |
| Number of complaints^b^ (t2) |  |  |  |  |  |  |  |  |
| 0 complaint (ref.) | 1.00 | - | 1.00 | - | 1.00 | - | 1.00 | - |
| 1 complaint | *1.90**** | 1.42; 2.56 | *1.70**** | 1.26; 2.29 | *1.67*** | 1.19; 2.34 | *1.63**** | 1.28; 2.07 |
| ≥2 complaints | 2.93*** | 1.91; 4.48 | 2.81*** | 2.13; 3.69 | 3.28*** | 2.16; 4.98 | 3.01*** | 2.29; 3.96 |
|  |  |  |  |  |  |  |  |  |
| Persistence of complaints^c^ |  |  |  |  |  |  |  |  |
| Neither at t1 nor at t2 (ref.) | 1.00 | - | 1.00 | - | 1.00 | - | 1.00 | - |
| At t1 but not at t2 | 2.24** | 1.34; 3.75 | *1.56** | 1.07; 2.29 | 2.16** | 1.27; 3.68 | *1.95**** | 1.38; 2.76 |
| At t2 but not at t1 | 2.80*** | 1.77; 4.43 | 1.99***** | 1.43; 2.77 | 3.12*** | 1.94; 5.02 | *2.16**** | 1.56; 2.99 |
| At t1 and t2 | 1.94 | 0.94; 4.04 | *2.80**** | 2.07; 3.80 | 2.58* | 1.24; 5.38 | 3.35***** | 2.43; 4.62 |

^a^ At least one psychosomatic complaint (stomach ache, headache and/or difficulties falling asleep). Figures in italics indicate statistically significant difference from the category “Weekly” (p<0.05).

^b^ Number of complaints more often than weekly (includes the “More often than weekly” and “Daily” categories). Figures in italics indicate statistically significant difference from the category “≥2 complaints” (p<0.05).

^c^ ≥2 psychosomatic complaints more often than weekly (includes the “More often than weekly” and “Daily” categories). Figures in italics indicate statistically significant difference from the category “At t1 and t2” (p<0.05).

***p<0.001 **p<0.01 *p<0.05

**Table A.5** Average marginal effects (AME) with 95% confidence intervals (CI) from binary logistic regression models analysing the associations between the frequency, number, and persistence of psychosomatic complaints at t1 and t2 and depression and anxiety symptoms at t3, stratified by gender. Models adjust for family type, parental education, and parental country of birth.

|  | Depression symptoms (t3) | | | | Anxiety symptoms (t3) | | | |
| --- | --- | --- | --- | --- | --- | --- | --- | --- |
|  | Males (n=1169) | | Females (n=1610) | | Males (n=1169) | | Females (n=1610) | |
|  | AME | 95% CI | AME | 95% CI | AME | 95% CI | AME | 95% CI |
| Frequency of complaints^a^ (t1) |  |  |  |  |  |  |  |  |
| Less often than weekly (ref.) | 0.00 | - | 0.00 | - | 0.00 | - | 0.00 | - |
| Weekly | 0.06 | -0.01; 0.13 | 0.03 | -0.03; 0.09 | 0.05 | -0.01; 0.10 | 0.04 | -0.03; 0.11 |
| More often than weekly | 0.13*** | 0.07; 0.20 | 0.07* | 0.01; 0.12 | 0.11*** | 0.05; 0.17 | *0.12**** | 0.06; 0.18 |
| Daily | 0.14** | 0.04; 0.23 | *0.20**** | 0.13; 0.28 | *0.15**** | 0.06; 0.24 | *0.28**** | 0.21; 0.36 |
|  |  |  |  |  |  |  |  |  |
| Frequency of complaints^a^ (t2) |  |  |  |  |  |  |  |  |
| Less often than weekly (ref.) | 0.00 | - | 0.00 | - | 0.00 | - | 0.00 | - |
| Weekly | 0.08* | 0.02; 0.15 | 19.1 | -0.002 | 18.4 | 0.07** | 0.12*** | 0.06; 0.18 |
| More often than weekly | 0.14*** | 0.08; 0.20 | 29.4 | *0.10**** | 20.7 | 0.09*** | 0.16*** | 0.11; 0.22 |
| Daily | *0.25**** | 0.15; 0.36 | 42.8 | *0.22**** | 35.9 | *0.24**** | *0.34**** | 0.27; 0.41 |
|  |  |  |  |  |  |  |  |  |
| Number of complaints^a^ (t1) |  |  |  |  |  |  |  |  |
| 0 complaint (ref.) | 0.00 | - | 0.00 | - | 0.00 | - | 0.00 | - |
| 1 complaint | 0.10** | 0.04; 0.16 | *0.06** | 0.01; 0.11 | 0.09** | 0.03; 0.15 | *0.10**** | 0.04; 0.15 |
| ≥2 complaints | 0.15** | 0.06; 0.25 | 0.15*** | 0.09; 0.21 | 0.14** | 0.05; 0.23 | 0.23*** | 0.17; 0.29 |
|  |  |  |  |  |  |  |  |  |
| Number of complaints^a^ (t2) |  |  |  |  |  |  |  |  |
| 0 complaint (ref.) | 0.00 | - | 0.00 | - | 0.00 | - | 0.00 | - |
| 1 complaint | 0.11*** | 0.06; 0.17 | *0.09*** | 0.04; 0.15 | *0.07*** | 0.02; 0.12 | *0.10**** | 0.05; 0.15 |
| ≥2 complaints | 0.20*** | 0.10; 0.29 | 0.19*** | 0.14; 0.25 | 0.20*** | 0.11; 0.28 | 0.24***** | 0.18; 0.31 |
|  |  |  |  |  |  |  |  |  |
| Persistence of complaints^a^ |  |  |  |  |  |  |  |  |
| Neither at t1 nor at t2 (ref.) | 0.00 | - | 0.00 | - | 0.00 | - | 0.00 | - |
| At t1 but not at t2 | 0.15** | 0.04; 0.26 | *0.08** | 0.00; 0.15 | 0.11* | 0.01; 0.22 | *0.15**** | 0.06; 0.23 |
| At t2 but not at t1 | 0.19*** | 0.09; 0.30 | 0.12** | 0.05; 0.19 | 0.19*** | 0.09; 0.29 | *0.17**** | 0.09; 0.25 |
| At t1 and t2 | 0.13 | -0.04; 0.29 | 0.21*** | 0.14; 0.28 | 0.17* | 0.01; 0.32 | 0.28***** | 0.20; 0.35 |

^a^ At least one psychosomatic complaint (stomach ache, headache and/or difficulties falling asleep). Figures in italics indicate statistically significant difference from the category “Weekly” (p<0.05).

^b^ Number of complaints more often than weekly (includes the “More often than weekly” and “Daily” categories). Figures in italics indicate statistically significant difference from the category “≥2 complaints” (p<0.05).

^c^ ≥2 psychosomatic complaints more often than weekly (includes the “More often than weekly” and “Daily” categories). Figures in italics indicate statistically significant difference from the category “At t1 and t2” (p<0.05).

***p<0.001 **p<0.01 *p<0.05

**Table A.6** Means, standard deviations and regression coefficients (b) with 95% confidence intervals (CI) from ordinary least squares (OLS) regression models analysing the associations between the frequency, number, and persistence of psychosomatic complaints at t1 and t2 and depression and anxiety symptoms at t3 (the outcomes are non-dichotomised variables with a range from 0 to 6), stratified by gender. Models adjust for family type, parental education, and parental country of birth.

|  | Depression symptoms (t3) | | | | | | Anxiety symptoms (t3) | | | | | |
| --- | --- | --- | --- | --- | --- | --- | --- | --- | --- | --- | --- | --- |
|  | Males (n=1169) | | | Females (n=1610) | | | Males (n=1169) | | | Females (n=1610) | | |
|  | M (SD) | b | 95% CI | M (SD) | b | 95% CI | M (SD) | b | 95% CI | M (SD) | b | 95% CI |
| Frequency of complaints^a^ (t1) |  |  |  |  |  |  |  |  |  |  |  |  |
| Less often than weekly (ref.) | 1.29(1.43) | 0.00 | - | 1.41(1.46) | 0.00 | - | 1.07(1.37) | 0.00 | - | 1.81(1.67) | 0.00 | - |
| Weekly | 1.67(1.62) | 0.40** | 0.16; 0.63 | 1.61(1.41) | 0.20 | -0.02; 0.42 | 1.44(1.56) | 0.38** | 0.16; 0.60 | 2.01(1.63) | 0.20 | -0.06; 0.46 |
| More often than weekly | 1.99(1.67) | 0.68*** | 0.45; 0.91 | 1.89(1.64) | *0.45**** | 0.25; 0.65 | 1.74(1.76) | 0.66*** | 0.42; 0.90 | 2.45(1.80) | *0.63**** | 0.40; 0.85 |
| Daily | 2.20(1.60) | *0.83**** | 0.51; 1.16 | 2.46(1.71) | *1.00**** | 0.76; 1.25 | 1.97(1.82) | *0.85**** | 0.47; 1.23 | 3.09(1.96) | *1.26**** | 0.97; 1.55 |
|  |  |  |  |  |  |  |  |  |  |  |  |  |
| Frequency of complaints^a^ (t2) |  |  |  |  |  |  |  |  |  |  |  |  |
| Less often than weekly (ref.) | 1.25(1.40) | 0.00 | - | 1.27(1.39) | 0.00 | - | 0.99(1.31) | 0.00 | - | 1.60(1.47) | 0.00 | - |
| Weekly | 1.62(1.56) | 0.38** | 0.13; 0.64 | 1.60(1.39) | 0.31** | 0.10; 0.52 | 1.53(1.63) | 0.54*** | 0.31; 0.76 | 2.17(1.75) | 0.54*** | 0.31; 0.77 |
| More often than weekly | 1.88(1.66) | 0.62*** | 0.39; 0.84 | 1.92(1.60) | 0.61*** | 0.41; 0.80 | 1.59(1.61) | 0.59*** | 0.37; 0.81 | 2.43(1.81) | *0.79**** | 0.59; 1.00 |
| Daily | 2.45(1.68) | *1.15**** | 0.81; 1.50 | 2.52(1.78) | 1.19*** | 0.94; 1.44 | 2.21(1.97) | *1.20**** | 0.82; 1.59 | 3.13(1.95) | *1.49**** | 1.22; 1.76 |
|  |  |  |  |  |  |  |  |  |  |  |  |  |
| Number of complaints^a^ (t1) |  |  |  |  |  |  |  |  |  |  |  |  |
| 0 complaint (ref.) | 1.41(1.50) | 0.00 | - | 1.49(1.44) | 0.00 | - | 1.18(1.43) | 0.00 | - | 1.89(1.66) | 0.00 | - |
| 1 complaint | 1.94(1.61) | *0.50**** | 0.29; 0.72 | *1.87(1.60)* | *0.35**** | 0.17; 0.53 | 1.78(1.72) | 0.57*** | 0.33; 0.80 | 2.38(1.79) | *0.48**** | 0.27; 0.69 |
| ≥2 complaints | 2.31(1.73) | 0.87*** | 0.52; 1.22 | 2.33(1.74) | 0.79*** | 0.58; 0.99 | 1.87(1.92) | 0.67** | 0.28; 1.05 | 2.99(1.93) | 1.08*** | 0.85; 1.30 |
|  |  |  |  |  |  |  |  |  |  |  |  |  |
| Number of complaints^a^ (t2) |  |  |  |  |  |  |  |  |  |  |  |  |
| 0 complaint (ref.) | 1.38(1.47) | 0.00 | - | 1.42(1.40) | 0.00 | - | 1.18(1.45) | 0.00 | - | 1.85(1.63) | 0.00 | - |
| 1 complaint | 1.88(1.66) | *0.48**** | 0.28; 0.68 | 1.85(1.57) | *0.41**** | 0.23; 0.60 | 1.63(1.68) | *0.44**** | 0.22; 0.65 | 2.33(1.79) | *0.46**** | 0.26; 0.66 |
| ≥2 complaints | 2.41(1.69) | 0.97*** | 0.64; 1.30 | 2.43(1.76) | 0.95*** | 0.75; 1.15 | 2.05(1.80) | 0.84*** | 0.50; 1.18 | 3.04(1.92) | 1.16*** | 0.93; 1.39 |
|  |  |  |  |  |  |  |  |  |  |  |  |  |
| Persistence of complaints^a^ |  |  |  |  |  |  |  |  |  |  |  |  |
| Neither at t1 nor at t2 (ref.) | 1.48(1.51) | 0.00 | - | 1.53(1.45) | 0.00 | - | 1.27(1.49) | 0.00 | - | 1.94(1.66) | 0.00 | - |
| At t1 but not at t2 | 2.20(1.80) | 0.68** | 0.25; 1.11 | 1.97(1.62) | *0.40*** | 0.13; 0.68 | 1.93(1.98) | 0.63** | 0.15; 1.10 | 2.70(1.87) | *0.75**** | 0.43; 1.07 |
| At t2 but not at t1 | 2.35(1.75) | 0.80*** | 0.43; 1.18 | 2.22(1.71) | *0.63**** | 0.38; 0.88 | 2.16(1.80) | 0.86*** | 0.47; 1.24 | 2.84(1.88) | *0.89**** | 0.60; 1.17 |
| At t1 and t2 | 2.53(1.57) | 1.05*** | 0.49; 1.60 | 2.61(1.78) | 1.03*** | 0.77; 1.29 | 1.75(1.80) | 0.50 | -0.13; 1.12 | 3.22(1.94) | 1.26*** | 0.97; 1.55 |

^a^ At least one psychosomatic complaint. Figures in italics indicate statistically significant difference from the category “Weekly” (p<0.05).

^b^ Number of complaints more often than weekly (includes the “More often than weekly” and “Daily” categories). Figures in italics indicate statistically significant difference from the category “≥2 complaints” (p<0.05).

^c^ ≥2 psychosomatic complaints more often than weekly (includes the “More often than weekly” and “Daily” categories). Figures in italics indicate statistically significant difference from the category “At t1 and t2” (p<0.05).

***p<0.001 **p<0.01 *p<0.05
